# Sostdc1 Secreted from Cutaneous Lymphatic Vessels Acts as a Paracrine Factor for Hair Follicle Growth

**DOI:** 10.3390/cimb44050146

**Published:** 2022-05-12

**Authors:** Sun-Young Yoon, Michael Detmar

**Affiliations:** 1 Department of Cosmetic Science, Kwangju Women’s University, Gwangju 62396, Korea; 2 Institute of Pharmaceutical Sciences, Swiss Federal Institute of Technology, ETH Zurich, 8093 Zurich, Switzerland

**Keywords:** lymphatic vessels, hair follicle growth, Sostdc1, dermal papilla cells

## Abstract

In our previous study, we found that lymphatic vessels stimulate hair follicle growth through paracrine effects on dermal papilla cells. However, the paracrine factors secreted from cutaneous lymphatic vessels that can activate dermal papilla cells are still unknown. In this study, we investigated whether lymphatic endothelial cells might secrete paracrine factors that activate dermal papilla cells in vitro. We found that Sostdc1 was more expressed in lymphatic endothelial cells compared with blood vascular endothelial cells. In addition, Sostdc1 expression levels were significantly increased during the anagen phase in the back skin of C57BL/6J mice, as compared to the telogen phase. We also observed that incubation of dermal papilla cells with 200 ng/mL Sostdc1 for 72 h induced the expression levels of Lef-1, a downstream target of Wnt signaling. Taken together, our results reveal that Sostdc1, a BMP antagonist, secreted from cutaneous lymphatic vessels, may act as a paracrine factor for hair follicle growth.

## 1. Introduction

Hair follicle (HF) is a small organ in the skin and consists of anagen (active growth stage), catagen (regression of the hair follicle stage), and telogen (resting stage) phases [1]. HF stem cells, which reside within the bulge region of the HF, promote the repetitive regeneration of the follicle during the hair cycle. During the telogen to anagen transition, HF stem cells receive stimulatory signals from dermal papilla cells (DPCs), and from the cutaneous microenvironment, including dermal fibroblasts, adipocytes, preadipocytes, blood vasculature, nerve fibers and macrophages [2,3,4]. In a previous study, we found that the production of vascular endothelial growth factor (VEGF)-A, a key regulator of angiogenesis, is up-regulated during the anagen phase, and VEGF-A-induced angiogenesis promotes hair follicle growth in mice [5]. The lymphatic vascular system consists of lymphatic capillaries, collecting lymphatic vessels, thoracic duct and lymph nodes and plays important roles in the control of tissue fluid homeostasis and immune responses [6,7,8,9]. VEGF-C, a lymphangiogenic growth factor, is the main driver of lymphangiogenesis via VEGF receptor-3 (VEGFR-3) [8,10,11,12]. It has been reported that lymphatic capillaries form intimate networks around HF stem cells and these HF stem cells undergo a secretome switch that causes transient dissociation of lymphatics for hair regeneration [13]. In addition, lymphatic vessels (LVs) as stem cell niche components have been shown to coordinate HF connections, and the depletion of LVs blocks HF growth in vivo [14]. Our previous study also showed that increased LV density in the skin of K14-VEGF-C transgenic mice prolonged anagen HF growth, while in K14-sVEGFR3 transgenic mice that lack cutaneous LVs, entry of HFs into the catagen phase was accelerated [15]. Our previous findings indicate that LVs directly connect the individual HFs and LVs and promote HF growth in transgenic mice with increased levels of cutaneous VEGF-C [15]. In addition, we previously reported that conditioned media of lymphatic endothelial cells (LECs) promote the growth of human DPCs and expression of IGF-1 and alkaline phosphatase, both activators of DPCs, suggesting that LVs stimulate hair growth through the paracrine effects on DPCs [15]. However, the molecular mechanisms by which lymphatic endothelium might activate DPCs are still unknown. In this study, we provided evidence that LECs secrete sostdc1 that activates DPCs. Taken together, our results reveal that LVs may stimulate HF growth through Sostdc1 secretion, indicating a potential therapeutic strategy for the treatment of hair loss.

## 2. Materials and Methods

### 2.1. Mouse Models

To check the expression levels of Sostdc1 on the hair cycle after depilation-induced hair regeneration, the back skin of 8-week-old female C57BL/6J mice in the telogen phase was depilated using wax as described [1,16], resulting in the synchronized induction of new anagen follicle growth. Mice were sacrificed on days 12 (anagen phase) and 22 (telogen phase) after depilation, and back skin tissue samples were collected for quantitative real-time PCR analysis. To examine the Sostdc1 mRNA expression levels on the postnatal hair cycle, back skin samples of C57BL/6J female mice were obtained at postnatal days 28 (anagen phase) and 49 (telogen phase). All experimental procedures were conducted according to animal protocols approved by the Kantonales Veterinaeramt Zuerich (protocol 237/2013).

### 2.2. Immunofluorescence Stainings

Back skin samples were embedded in OCT (Leica Biosystems, Newcastle, UK) and frozen in liquid nitrogen. Further, 10-μm frozen sections were fixed in 4% paraformaldehyde for 15 min at room temperature (RT), washed in PBS and incubated with blocking solution (5% donkey serum, 0.2% BSA and 0.3% Triton X-100 in PBS) for 1 h at RT. Next, the sections were stained with goat anti-Prox1 (R&D systems, Minneapolis, MN, USA) and rabbit anti-Sostdc1 (Abcam, Cambridge, MA, USA) antibodies overnight at 4 °C. After several washes, the sections were then incubated with Alexa Fluor 488 donkey anti-goat (Thermo Fisher Scientific, San Jose, CA, USA) and Alexa Fluor 594 donkey anti-rabbit (Thermo Fisher Scientific) antibodies for 30 min at RT. Hoechst 33342 (Invitrogen, Carlsbad, CA, USA) was used for nuclear staining. Immunofluorescence images were acquired with a Zeiss LSM 710 FCS confocal microscope (Carl Zeiss, Jena, Germany).

### 2.3. Cell Culture

Human DPCs (ScienCell Research Laboratories, Carlsbad, CA, USA) were maintained in Dulbecco’s Modified Eagle’s Medium (DMEM; Gibco, Grand Island, NY, USA) supplemented with 10% fetal bovine serum (FBS; Gibco), 10 ng/mL basic fibroblast growth factor (Peprotech, London, UK) and antibiotic/antimycotic solution (Gibco). Primary human LECs and blood vascular endothelial cells (BECs) isolated from foreskin were cultured as described previously [17]. LECs were incubated on 10 μg/mL fibronectin-coated dishes in endothelial cell basal medium (EBM; Lonza, Walkersville, MD, USA) containing 20% FBS, 2 mM l-glutamine (Gibco), antibiotic-antimycotic solution, 10 μg/mL hydrocortisone (Sigma-Aldrich, St. Louis, MO, USA) and 25 μg/mL cAMP (Sigma-Aldrich). BECs were incubated on 10 μg/mL fibronectin-coated dishes in EBM supplemented with 20% FBS, 2 mM l-glutamine, antibiotic-antimycotic solution and 0.4% endothelial cell growth supplement (ECGS; PromoCell, Heidelberg, Germany). Cells were incubated at 37 °C and 5% CO_2_ in a humidified incubator.

### 2.4. Quantitative Real-Time Polymerase Chain Reaction (qRT-PCR)

Total RNA was isolated from DPCs and LECs using NucleoSpin RNA (Macherey-Nagel, Düren, Germany) and then treated with DNase to remove genomic DNA. An amount of 1 µg of total RNA was used to synthesize cDNA with the High Capacity Reverse Transcription kit (Applied Biosystems, Foster City, CA, USA). PCR was conducted on a 7900HT Fast Real-Time PCR System (Applied Bio-systems, Waltham, MA, USA) using FastStart SYBR green master mix (Roche Diagnostics, Basel, Switzerland) according to the manufacturer’s instructions. Gene expression levels were normalized to levels of the control gene, Rplp0 (36B4). Primer information is shown in Table 1.

### 2.5. Enzyme-Linked Immunosorbent Assay (ELISA)

To examine the Sostdc1 secretion, conditioned media of LECs were collected. When LECs reached 80% confluence, the growth medium was replaced with EBM containing 2% FBS. Conditioned media of LECs were collected after 48 h and centrifuged. For the preparation of conditioned media of control, EBM containing 2% FBS was added to empty cell culture dishes and collected after 48 h. The amount of Sostdc1 in conditioned media of LECs was determined with a Sostdc1 ELISA kit (Biomatik, Wilmington, DE, USA) according to the manufacturer’s instructions. Measurement of the absorbance was performed at 450 nm using a microplate reader.

### 2.6. Knockdown of Sostdc1

LECs were infected with adenoviral Sostdc1 shRNA (multiplicity of infection; MOI = 50, Vector BioLabs, Philadelphia, PA, USA) or scrambled shRNA (MOI = 50, Vector BioLabs, Philadelphia, PA, USA) as a control. After 24 h, the growth medium was replaced with EBM containing 2% FBS. Conditioned media of Sostdc1 shRNA treated LECs (LEC-CM-Sostdc1 shRNA) or scrambled shRNA treated LECs (LEC-CM-scrambled shRNA) were collected after 24 h and centrifuged. DPCs were incubated with 50% LEC-CM-Sostdc1 shRNA or LEC-CM-scrambled shRNA for 48 h.

### 2.7. Statistical Analyses

Statistical significance (*p* < 0.05) was determined using a two-tailed unpaired *t*-test (GraphPad Software, San Diego, CA, USA). All experiments were performed independently three times.

## 3. Results and Discussion

### 3.1. Sostdc1 Were Highly Expressed in Lymphatic Endothelial Cells

HF stem cells have been shown to be activated by DPCs and the perifollicular microenvironment, such as dermal fibroblasts, adipocytes, preadipocytes, macrophages and the blood vasculature [2,3,4,5,18]. DPCs are composed of specialized mesenchymal cells at the bottom of the HF and play important roles in hair formation, anagen induction and the maintenance of HF development [19,20,21,22]. In our previous findings, dermal LVs connect the individual HFs and are located in close proximity to the DPCs and the stem cell area of murine HFs [15]. In addition, LVs stimulate HF growth in mice and the conditioned media of LECs increase the proliferation of human DPCs and expression of IGF-1 and alkaline phosphatase, indicating that LVs promote hair growth through paracrine effects on DPCs [15].

**Figure 1:**
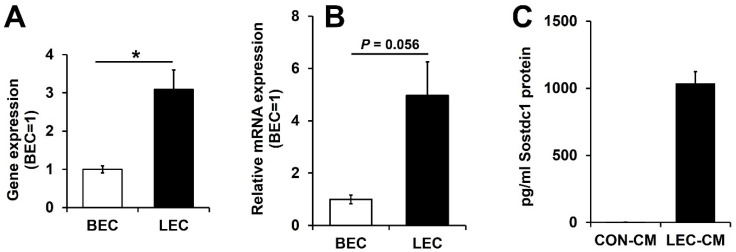
Sostdc1 is highly expressed in lymphatic endothelial cells compared with blood vascular endothelial cells. (**A**,**B**) The expression of Sostdc1 in matched pairs of human LECs and BECs was determined by microarray (**A**) and by qRT-PCR (**B**). (**C**) Sostdc1 protein levels were measured by ELISA in conditioned media of control (CON-CM) and conditioned media of LECs (LEC-CM). Results are expressed as mean ± standard error of the mean (SEM). Data were analyzed using a two-tailed unpaired *t*-test. * *p* < 0.05 compared to the control group.

**Figure 2:**
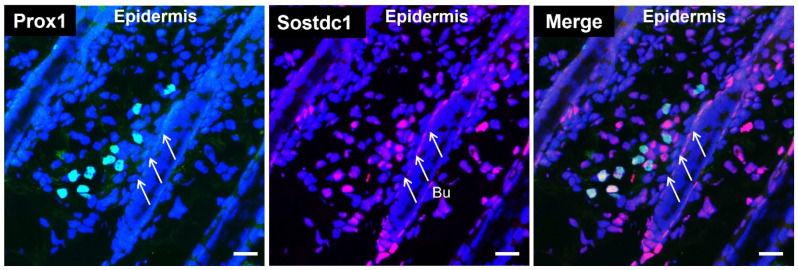
Lymphatic vessels express Sostdc1. Immunofluorescent staining of 10-µm frozen sections of back skin (anagen phase, postnatal day 8) for Prox1 (lymphatic-specific transcription factor, green) and Sostdc1 (red). Nuclear staining with Hoechst 33342 (blue). White arrows indicate the bulge area (Bu). Scale bars: 20 μm.

To investigate whether LVs might secrete paracrine factors that activate DPCs, we analyzed the transcriptomes of matched pairs of cultured human dermal LECs and BECs that we have previously established (GEO accession: GSE6549) for hair cycle-related genes with higher expression in lymphatic endothelial cells. We found that LECs strongly express BMP inhibitors, including Noggin, Sostdc1 and TMEFF1. BMPs, such as BMP2 and BMP4, have been reported to inhibit HF growth, whereas BMP inhibitors attenuate the inhibitory effect of BMPs and activate Wnt signaling [3,4,18,23,24,25]. Among these BMP inhibitors, Sostdc1 was more highly expressed in LECs in the array data (Figure 1A). Sostdc1 has been shown to be expressed in DPCs and hair matrix keratinocytes, and the lack of Sostdc1 in Sox2 knockout mice increased BMP activity and decreased migration during hair shaft differentiation [26]. Quantitative RT-PCR analysis confirmed that Sostdc1 mRNA expression levels were higher in LECs than in BECs (Figure 1B). To investigate the Sostdc1 secretion, the amount of Sostdc1 was determined by ELISA analysis. We found that conditioned media of LECs contained up to 1 ng/mL of Sostdc1, indicating that LVs secreted Sostdc1 (Figure 1C). To further characterize the Sostdc1 expression of LVs, we performed double immunofluorescence stainings for Sostdc1 and lymphatic-specific transcription factor Prox1^+^. We found that LVs expressed Sostdc1, indicating that Sostdc1 secreted from cutaneous LVs may act as a paracrine factor for HF growth (Figure 2).

### 3.2. Sostdc1 Expression Levels Were Significantly Increased during the Anagen Phase

**Figure 3:**
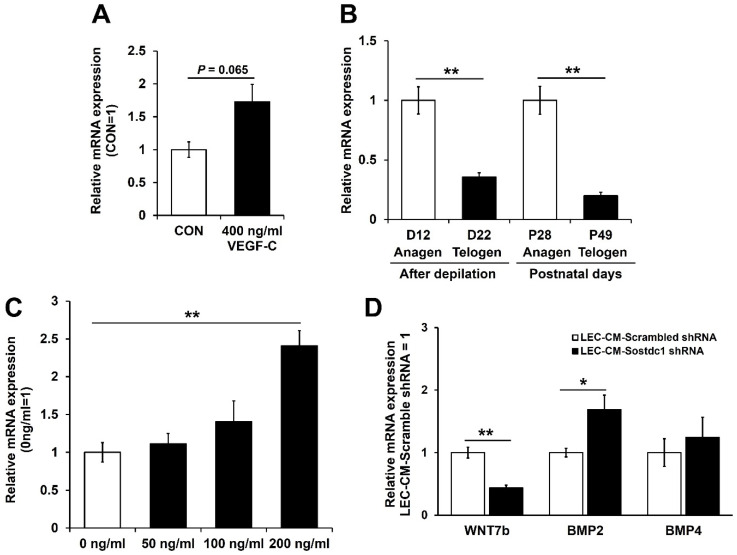
Sostdc1 expression levels were significantly increased during the anagen phase. (**A**) LECs were incubated with 400 ng/mL VEGF-C for 24 h, followed by qRT-PCR to assess Sostdc1 expression. (**B**) Total RNA was isolated from the back skin of female C57BL/6 mice and Sostdc1 expression was determined by qRT-PCR. (**C**) DPCs were treated with the indicated concentrations of Sostdc1 for 72 h and Lef-1 mRNA expression was determined by qRT-PCR. (**D**) DPCs were incubated with 50% LEC-CM-Sostdc1 shRNA or LEC-CM-scrambled shRNA for 48 h. Total RNA was isolated and mRNA expression levels of WNT7b, BMP2 and BMP4 were measured by qRT-PCR. Results are expressed as mean ± SEM. Data were analyzed using a two-tailed unpaired *t*-test. * *p* < 0.05, ** *p* < 0.01 compared to the control group.

VEGF-C, a lymphangiogenic growth factor, is the major driver of lymphangiogenesis via VEGFR-3 [11,12], and our previous findings indicate that LVs promote HF growth in transgenic mice with increased levels of VEGF-C [15]. It is of interest that incubation of LECs with 400 ng/mL of VEGF-C for 24 h resulted in increased Sostdc1 mRNA expression (Figure 3A). Next, we investigated whether the expression levels of Sostdc1 might undergo cyclic changes during the hair cycle. Importantly, during depilation-induced hair regeneration, the mRNA expression levels of Sostdc1 in the back skin of female C57BL/6J mice were significantly increased during the anagen phase (at day 12 after depilation), as compared with the telogen phase (at day 22 after depilation) (Figure 3B). Similarly, during the postnatal hair cycle, Sostdc1 mRNA expression levels were significantly higher during the anagen phase (P28) than during the telogen phase (P49) (Figure 3B). In the next step, DPCs were treated with 50, 100 or 200 ng/mL recombinant Sostdc1 protein for 72 h. Wnt signaling is required for the development of the HFs, and Lef-1 is a downstream target of Wnt signaling [3,27]. After treatment with 200 ng/mL of Sostdc1, Lef-1 expression was markedly enhanced in DPCs (Figure 3C). These results reveal that Sostdc1, a BMP antagonist, was secreted by LECs and activates DPCs, indicating LVs may stimulate HF growth through Sostdc1 secretion. To further investigate the effects of LEC-secreted Sostdc1 on DPCs, we performed a knockdown of Sostdc1. LECs were infected with adenoviral Sostdc1 shRNA or scrambled shRNA as a control. After 24 h, the growth medium was replaced with EBM containing 2% FBS. Conditioned media of Sostdc1 shRNA treated LECs (LEC-CM-Sostdc1 shRNA) or scrambled shRNA treated LECs (LEC-CM-scrambled shRNA) were collected after 24 h and centrifuged. DPCs were incubated with 50% LEC-CM-Sostdc1 shRNA or LEC-CM-scrambled shRNA for 48 h. Incubation of DPCs with LEC-CM-Sostdc1 shRNA markedly decreased the mRNA expression levels of WNT7b, a stimulatory factor of hair growth [3,28], and increased BMP2 expression, as compared to DPCs incubated with LEC-CM-scrambled shRNA (Figure 3D). No major changes were found regarding the expression of BMP4 mRNA (Figure 3D). Together, these results indicate that lymphatic endothelium might contribute to DPC activation via secretion of Sostdc1. Further studies would be of interest to investigate the distinct biological role of LEC-derived Sostdc1 in hair cycling using lymphatic-specific Sostdc1 knockout mice.

## 4. Conclusions

Previously, we found that dermal LVs reside in close proximity to the DPCs and the stem cell regions of murine HFs, and stimulate HF growth through paracrine effects on DPCs [15]. However, the paracrine factors secreted from the LVs that can activate DPCs have remained unknown. In this study, we found that Sostdc1 was secreted by LECs, and mRNA expression levels of Sostdc1 in C57BL/6J mice were significantly induced during the anagen phase, as compared with the telogen phase. In addition, incubation of DPCs with 200 ng/mL of Sostdc1 markedly increased Lef-1 expression. Taken together, LVs may contribute to the stimulation of HF growth through Sostdc1 secretion, indicating a potential therapeutic strategy for the treatment of hair loss.

## Figures and Tables

**Table 1:** Sequences of primers for human genes.

Gene Name	RefSeq (NCBI Database)	Primer Sequence	Cycles
RPLP0	NM_053275		
forward		CAG ATT GGC TAC CCA ACT GTT	40
reverse		GGG AAG GTG TAA TCC GTC TCC	
Lef-1	NM_001130714		
forward		GGG TGG TGT TGG ACA GAT CA	40
reverse		TGT CAG TGT GGG GAT GTT CC	
Sostdc1	NM_015464		
forward		GGA TTG GAG GAG GCT ATG GA	40
reverse		TTT TCC GCT CTC TGT GAT GC	
WNT7b	NM_058238		
forward		CTG GGA GCC AAC ATC ATC TG	40
reverse		AGT TGC TCA GGT TCC CTT GG	
BMP2	NM_001200		
forward		AAC GAG TGG GAA AAC AAC CC	40
reverse		GTC ACG GGG AAT TTC GAG TT	
BMP4	NM_001347914		
forward		CAC TGG CTG ACC ACC TCA AC	40
reverse		GGC ACC CAC ATC CCT CTA CT	

## Data Availability

Not applicable.
